# Solvent-Impregnated Sorbents for Tantalum from Niobium Separation Using a Fixed-Bed Column

**DOI:** 10.3390/ma15041513

**Published:** 2022-02-17

**Authors:** Magdalena Turkowska, Krzysztof Karoń, Andrzej Milewski, Agata Jakóbik-Kolon

**Affiliations:** 1 InorChem Centre for Research and Development, Łukasiewicz Research Network New Chemical Syntheses Institute, Sowińskiego 11, 44-101 Gliwice, Poland; 2 Faculty of Chemistry, Silesian University of Technology, Krzywoustego 6, 44-100 Gliwice, Poland; krzysztof.karon@polsl.pl (K.K.); andrzej.milewski@polsl.pl (A.M.); agata.jakobik@polsl.pl (A.J.-K.)

**Keywords:** niobium, tantalum, separation, activated carbon, methyl isobutyl ketone

## Abstract

Reactor-grade niobium steel is used as a construction material for nuclear reactors. In this case, the presence of tantalum, which is characterized by a 20 times higher active cross section for capturing thermal neutrons than the cross section of niobium (^181^Ta: 21.3 barn), cannot exceed 100 ppm. Analytical methods for quality and new separation method development control need very pure niobium matrices—niobium compounds with a low tantalum content, which are crucial for preparing matrix reference solutions or certified reference materials (CRMs). Therefore, in this paper, a new, efficient method for separating trace amounts of Ta(V) from Nb(V) using extraction chromatography with the use of sorbents impregnated with methyl isobutyl ketone MIBK solvent is proposed. Various types of MIBK-impregnated sorbents were used (AG^®^ 1-X8 Anion Exchange Resin, AMBERLITE™ IRC120 Na Ion Exchange Resin, SERVACEL^®^ Cellulose Anion Exchangers DEAE 52, active carbons of various grain size, carbonized blackcurrant pomace, carbonized chokeberry pomace, bentonite, and polyurethane foam in lumps). The highest tantalum removal efficiency was determined using active coal-based materials (>97%). The separation effectivity of tantalum from niobium was also determined in dynamic studies using a fixed-bed column with MIBK-impregnated active carbon. Solutions of various Nb:Ta weight ratios (1:1, 100:1, 1000:1) were used. The most impressive result was obtaining 70 mL of high purity niobium solution of tantalum content 0.027 ppm (in relation to Nb) with 88.4% yield of niobium from a solution of Nb:Ta, weight ratio 1000:1 (purge factor equaled 35,000). It proves the presented system to be applicable for preparation of pure niobium compounds with very low contents of tantalum.

## 1. Introduction

Niobium (Nb) and tantalum (Ta) are extremely important for a number of industrial branches, including the automotive, aviation, and space, construction, military, nuclear, electronic, and chemical industries [1,2] as well as for implantology [3]. Due to their excellent physical and chemical properties (high melting point, very high heat, and electric conductivity), as well as due to the lack of comparable substitutes in terms of pricing, Nb and Ta are often considered “strategic raw materials” [4,5]. Niobium is a key component of high strength low-alloy steel (HSLA) and addition of Nb in some parts per million strengthens this steel without deterioration of its plasticity. The production of HSLA constitutes almost 90% of the global use of Nb [6,7]. The remaining market for niobium [8,9] involves the production of superconducting magnets, cladding for nuclear fuel, medical implants, electronic components, optical lenses, and refractory metal superalloys [10]. The global production of niobium is 100,000 t per year, while the production of tantalum ranges from 500 to 2000 t per year due to of its lower quantity in nature [11]. The major application of tantalum is the production of electronic capacitors (60% of annual production of tantalum is used for the production of powder for capacitors) and specialized alloys for cutting tools, implants, military ammunition, and plane parts. Despite their strategic applications, Nb and Ta remain sparsely known elements to the majority of the public, and relatively little attention is paid to processing these two elements [11]. 

Technologically advanced applications of niobium usually require input materials with a purity of at least 99.5 wt.%, while tantalum concentrates have market value if the content of Ta is 20 wt.%. In all these raw materials, niobium is always present with tantalum and other elements, such as titanium, tungsten, zirconium, molybdenum, and hafnium [12]. The presence of these dopants has a negative effect on the mechanical properties of metallic niobium. In the case of niobium steel applied as construction materials for nuclear reactors, the presence of tantalum, which is characterized by an active cross section 20 times higher for capturing thermal neutrons than the cross section of niobium (^181^Ta: 21.3 barn), can lead to the formation of unfavorable long-life radioactive isotopes. Therefore, it is critical to lower the content of Ta and other impurities in niobium materials below 100 ppm (so-called reactor grade). The biggest problem is the purification and separation of tantalum from niobium, as both of these elements belong to Group 5, have an almost identical atomic and ionic radius (as a result of the contraction of lanthanide), and behave similarly [13,14]. However, in some cases, they show subtle differences in properties, which can be used to separate them using extraction methods, the ion-exchange method, adsorption methods, or fractional distillation of volatile chlorides. 

Currently, all the most important and commercial-processing methods involve the fluorination of Nb(V) and Ta(V) in their ores [15,16] or high-quality Nb-Ta concentrates during dissolution using fluoride agents, such as HF, HF with the addition of another mineral acid, HNO_3_/H_2_SO_4_ [17], or molten salts, which are the source of fluoride ions NH_4_F·HF [18,19], KF, and KF-HF [20]. Differences in the solubility of fluoride anion complexes of Nb(V) and Ta(V) in various organic solvents are utilized in solvent extraction in a liquid-liquid system, which was developed by Ames Laboratory and the U.S. Bureau of Mines in 1957 [21]. 

In HF solutions with a concentration of >35%, the ion [NbOF_5_]^2−^ is dominant, while for lower HF concentrations, this ion is conversed to [NbF_6_]^−^. For Ta(V), low concentrations of HF lead to the dominance of [TaF_7_]^2−^, while higher concentrations favor the formation of [TaF_6_]^−^. The differences in the dissociation constants and the formation constants, relevant acids H_2_TaF_7_ and H_2_NbOF_5_ are responsible for the separation of Nb(V) and Ta(V) (the formation constant of H_2_TaF_7_ is higher than the formation constant of H_2_NbOF_5_) [14,21]. 

The majority of the works were dedicated to the application of inert solvents for the extraction and separation of Nb(V) and Ta(V) from acid media containing HF, where the selective extraction of Ta(V)/Nb(V) was achieved by controlling the concentration of HF and the concentration of mineral acids and the concentration of metals [22,23,24]. Solvents such as: tributylphosphate (C_4_H_9_)_3_PO_4_ (TBP), n-octyl alcohol C_8_H_18_O (OCL), and methyl isobutyl ketone C_6_H_12_O (MIBK) [22,25] have been applied for separation and purification of Nb(V) and Ta(V) from HF solutions in the presence of H_2_SO_4_ or HCl, whereas no commercial application was reported for amines [22]. 

In the extraction and separation of Ta(V) and Nb(V) from fluoride solutions, MIBK is most frequently applied in the extraction and separation of Ta(V) and Nb(V) due to its high selectivity for Ta(V) and Nb(V), low density (0.802 mg/L), and viscosity (0.58 cP at 20 °C) [26]. For hydrofluoric acid HF, the most efficient separation was obtained when the concentration of free hydrofluoric acid (constituting the difference between the total concentration of HF in composition and the concentration of F^−^ in all Nb(V) and Ta(V) fluoride complexes) was minimum [27]. A low percent of Nb(V) extraction was the result of hydrolysis of H_2_NbF_7_ to H_2_NbOF_5_ that was not extracted with MIBK [28]. In systems such as HF + HNO_3_ or HF + H_2_SO_4_, the selective extraction of Ta(V)/Nb(V) using MIBK was observed for the concentration of mineral acids to be <8 M [28,29]. The efficiency of extracting Nb(V) from HF + H_2_SO_4_ using MIBK was better for the high ratio of the organic phase to aqueous one (O/A > 4), whereas the efficiency of extracting Ta(V) was hampered under such conditions. The effect of H_2_SO_4_ in fluoride solution on the extraction of Ta(V) and Nb(V) by MIBK was related to the salt effect [23,30]. 

However, the method of conventional liquid-liquid extraction (LLE) is economically and ecologically unfavorable, as it generates toxic waste due to the use of a large amount of solvents and their losses resulting from volatility or partial blending with the aqueous phase. The application of impregnated sorptive materials containing the relevant organic solvent for metal extraction with the use of reverse chromatography [31,32]—RPEC (Reversed Phase Extraction Chromatography)—is the solution to these problems. The RPEC uses the difference between the coefficient of two non-blending phases, namely the stationary phase, which is the extraction solvent placed on the porous hydrophobic medium, and the mobile phase, which is the solution of acid, base, or salt. Organic polymers, cross-linked styrene copolymers, solvent-impregnated resins, cross-linked copolymers of styrene polyurethane foam (PUF), as well as silica gel, diatomaceous earth, or powdered cellulose can be the medium for the stationary phase. Newly developed organic solvent carriers, such as bispropylurea bridged polysilsesquioxane [33], may contribute even more to this technique. The RPEC method can be implemented in a column or thin-layered variant where separation is carried out on a thin layer of sorbent placed on the bed made of glass, plastics, and aluminum. In such extraction systems, losses of organic solvent are strongly limited by immobilization, thanks to which corrosion problems of apparatuses are minimized, and economic as well as ecological considerations are taken into account. 

The RPEC method was used for sorption and separation of Nb(V) and Ta(V) on Amberlite XAD-7 impregnated with MIBK [34], Amberlite XAD-7 impregnated with p-tert-butylosulfinylocalix[4]arene (SOCA) [35], Kel-F^®^ resin (PCTFE polychlorotrifluoroethylene, Daikin Chemical Europe GmbH, Düsseldorf, Germany) impregnated with MIBK or DIPK [36], MIBK-saturated Teflon-6 [37], bis (2-ethyhexyl)phosphorus HDEHP placed on silica medium [38], polyurethane foam PUF impregnated with DAM, TBP, and MIBK [32].

It should be emphasized that in a majority of the separation cases described in the literature, solutions containing the comparable amounts of Nb(V) and Ta(V), or a little excess of one element with regard to the other one, was subjected to separation. These methods mainly concern the separation of the elements in question from their ores and their subsequent separation; however, the level of purification is not very deep (purity not greater than 99% [18]), but is generally lower [39,40]. However, for many currently applied separation methods, their efficiencies for a high initial ratio with respect to the content of niobium to tantalum, i.e., for separation of lower and lower contents of tantalum from niobium, are of great interest. This data is generally unavailable, but it constitutes crucial criteria for evaluating the method for its usability in obtaining high purity products.

The objective of this work was to develop and investigate the efficiency of separating trace amounts of Ta(V) from Nb(V) using extraction chromatography with the use of sorbents impregnated with MIBK solvent, and to propose a new separation system for the preparation of niobium compounds with low tantalum content. These studies are intended to show the possibility of obtaining very pure niobium matrices, namely niobium compounds with a low Ta content, which are crucial for preparing matrix reference solutions or certified reference materials (CRMs). This should decrease the limit of quantification (LOQ) of tantalum in niobium using a direct instrumental method, namely ICP-OES, for the quantification level of LOQ ≤ 10^−4^% Ta per Nb, which is currently impossible due to the lack of availability of these products on the market [41,42].

## 2. Materials and Methods

### 2.1. Materials and Reagents

The following sorbents were purchased: AG^®^ 1-X8 Anion Exchange Resin, analytical grade, 100 ÷ 200 mesh, chloride form (Bio-Rad, Hercules, CA, USA); AMBERLITE™ IRC120 Na Ion Exchange Resin; SERVACEL^®^ Cellulose Anion Exchangers DEAE 52 (Heidelberg, Germany); active carbon of grain size 0.5 ÷ 0.75 mm (Merck, Darmstadt, Germany; pulverized activated carbon p.a. (Avantor, Gliwice, Poland), carbonized blackcurrant pomace, carbonized chokeberry pomace, bentonite, and polyurethane foam in lumps.

The following reagents and solutions were used: hydrofluoric acid (47 ÷ 51% Normatom^®^, VWR, Radnor, PA, USA); nitric acid (67 ÷ 69% Normatom^®^, VWR, Radnor, PA, USA); hydrogen peroxide (30% Normapure^®^, VWR, Radnor, PA, USA); ammonia solution (28% Normapure^®^, VWR, Radnor, PA, USA); methyl isobutyl ketone MIBK (99%, Merck, Darmstadt, Germany); diammonium oxalate monohydrate aqueous solution 4% (analytical grade, Avantor, Gliwice, Poland); fluoride standard solution (1000 mg/L, Merck, Darmstadt, Germany); methyl orange (Avantor, Gliwice, Poland) aqueous solution 0.1%; buffer solution (TISAB II, Merck, Darmstadt, Germany); and Nb(V) oxide (Nb_2_O_5_, Specpure, Johnson-Matthey, London, England; Nb_2_O_5_, 99.99%, Acros Organics, Geel, Belgium) Ta(V) oxide (Ta_2_O_5_, Specpure, Johnson-Matthey, London, England) were used to prepare solutions of various Nb:Ta weight ratios. Calibration solutions were prepared from commercially available ICP tantalum and niobium standards traceable to SRM from NIST (concentration 1000 mg/mL in traces HNO_3_ and HF, Inorganic Ventures, Christiansburg, VA, USA). Argon of special purity for spectroscopy 99.999% (Technogas, Gliwice, Poland) and ultrapure water (ASTM Class 1, <18 MU cm^−1^) were also used.

### 2.2. Apparatus

Based on our previous studies of tantalum determination in the niobium matrix [41,42], depending on the tantalum content in niobium, Ta determination was performed using a Varian 810-MS inductively coupled plasma mass spectrometer (ICP-MS) (Varian, Palo Alto, CA, USA) (<3 ppm Ta in Nb) or a iCAP 7400+ MFC Duo inductively coupled plasma atomic emission spectrometer (ICP-AES) (Thermo) (>3 ppm Ta in Nb) equipped with a Duo EMT Torch Kit, cyclonic spray chamber, and sea spray nebulizer. The standards of tantalum with proper niobium matrix concentration were used. The niobium compound used as the matrix was purified from Ta by solvent extraction [42]. The parameters for tantalum analysis using the ICP-AES apparatus were as follows: RF power 1.35 kW, plasma flow 12 L/min, coolant gas flow 14 L/min, auxiliary gas flow 0.5 L/min, nebulizer gas flow 0.4 L/min, pump rate 80 rpm, and emission lines λ_Ta_ = 233.1, 240.0, and 263.5 nm. The parameters for tantalum analysis using the ICP-MS apparatus equipped with a micromist nebulizer, quartz Scott spray chamber (3 °C), platinum sampler cone, and nickel skimmer cone were as follows: RF power: 1.4 kW; plasma gas flow (argon): 17 L/min; auxiliary gas flow (argon): 1.7 L/min; nebulizer gas flow (argon): 1.00 L/min; pump rate: 4 rpm; sheath gas flow (argon): 0.2 L/min; and number of scans: 10, *m*/*z*: 181. Niobium content was determined by ICP-AES and the parameters were as follows: RF power: 1.35 kW; plasma flow: 12 L/min; coolant gas flow: 14 L/min; auxiliary gas flow: 0.5 L/min; nebulizer gas flow: 0.4 L/min; pump rate: 80 rpm; and emission lines: λ_Nb_ = 309.4, 313.0, 316.3, and 319.4 nm. The concentration of fluoride ions was measured using an ion meter (720 A, Thermo Electron Orion, Thermo Fisher Scientific Inc., Waltham, MA, USA).

### 2.3. Feed Solution Preparation

Nb-Ta solutions were prepared using a modular high pressure microwave digestion system (Multiwave 3000, Anton Paar, Graz, Austria) as described earlier [42]; briefly: The Nb and Ta pentoxide mixtures were digested with concentrated HF and HNO_3_ acids (7:4 *v*/*v*) using high-pressure microwave system to obtain the sample of the requested Nb:Ta weight ratio (1:1 and 100:1); for samples of lower Nb:Ta ratio (1000:1) a standard tantalum solution was used instead of the solid Ta compound. The solutions were then evaporated, almost to dryness (the precipitate should stay slightly wet), to remove free fluoride ions, and diluted with water to proper volume. The solution should contain trace amounts of fluoride ions (0.001 ÷ 0.01%).

### 2.4. Solvent-Impregnated Coals Preparation and Characterization

Coals (active carbon of grain size 0.5 ÷ 0.75 mm (C1), pulverized activated carbon (C2), and carbonized fruit (blackcurrant (C3) and chokeberry (C4)) pomace, was immersed with MIBK using the vacuum method and then the sorbent was left to achieve air-dry condition and placed in sealed container. Furthermore, some other sorption materials (strong base anion exchange resin (S1), strong acid cation exchange resin (S2), bentonite (S3), cellulose ion exchanger (S4), and polyurethane (PUR) foam in lumps (1 ÷ 2 mm) (S5) were solvent-loaded in the same way.

The carbon-based sorbents, prior the solvent load, were analyzed using a scanning electron microscope (Phenom Pro Desktop SEM—Phenom-World B.V., Eindhoven, The Netherlands) equipped with an EDS detector.

### 2.5. Preliminary Sorption Studies (Batch Mode)

A 3.5 g of solvent-impregnated sorbent was shaken with 50 mL of solution containing 7500 mg/L Nb and 1.335 mg/L Ta (178 ppm of Ta in relation to Nb) for 24 h, at room temperature (22 ± 1 °C). Then the sorbent was separated from the solution and the concentration of examined metal ions in the remaining solution (c) was measured using the ICP-AES apparatus. Based on the measured value, the removal efficiency (E (%)) was calculated using the following Equation (1):

(1) $$E=\frac{\left[c_{0}-c\right]}{c_{0}}\times 100\%$$

where:

c_0_—the initial concentration of metal ions in the solution (mg/L)

c—the final concentration of metal ions in the solution (mg/L).

A separation factor (SF), defined as the ratio of the tantalum partition coefficient (D_Ta_) to the niobium partition coefficient (D_Nb_) in the studied system, was introduced to evaluate the separation potential of the studied metals.

(2) $$\mathrm{SF}=\frac{D_{\mathrm{Ta}}}{D_{\mathrm{Nb}}}=\frac{E_{\mathrm{Ta}}\times \left[100-E_{\mathrm{Nb}}\right]}{E_{\mathrm{Nb}}\times \left[100-E_{\mathrm{Ta}}\right]}$$

where:

D_Nb_, D_Ta_—partition coefficient of Nb and Ta in the studied system

E_Nb_, E_Ta_—removal efficiency of Nb and Ta (%).

### 2.6. Separation of Tantalum from Niobium Using Fixed-Bed Column

The feed solution of Nb and Ta (prepared according to Section 2.3) was pumped through a column filled with 3.5 g of solvent-impregnated coal (C1) (bed height 18 cm, diameter 0.8 cm) by peristaltic pump with a constant flow rate of 0.2 mL/min. The fixed-bed columns were pretreated with a hydrofluoric acid solution of concentration 0.004%. Column effluent samples were collected in fractions of 10 mL. Tantalum and niobium contents in fractions were determined by ICP-AES or ICP-MS methods (Section 2.2). Experiments for all three initial Nb:Ta ratios were performed in duplicate, and the results presented are the mean values.

### 2.7. Stripping Tantalum from Solvent-Impregnated Active Coal (Batch Mode)

Approximately 3 g of solvent-impregnated active coal was contacted with a Nb and Ta ion solution of various Nb:Ta weight ratios (t = 24 h, V = 30 mL). The sorbent was then filtered and gently drained using filter paper and mixed for 15 min with a 40 mL solution of 4% ammonium oxalate or 15% hydrogen peroxide. The stripping procedure was repeated five times; for each step a fresh portion of stripping solution was used. The concentration of tantalum in the obtained solutions (after sorption and after desorption) was determined using the ICP-AES method. Standards of the same concentration of a stripping agent were used to eliminate matrix effects [42]. The desorption efficiency (DE (%)) was calculated using the Equation (3):

(3) $$\mathrm{DE}=\frac{m_{D}}{m_{s}}\times 100\%$$

where:

m*_D_*—the amount of tantalum ions desorbed (mg)

m*_s_*—the amount of tantalum ions loaded (mg).

## 3. Results

### 3.1. Carbons Characterization

**Figure 1:**
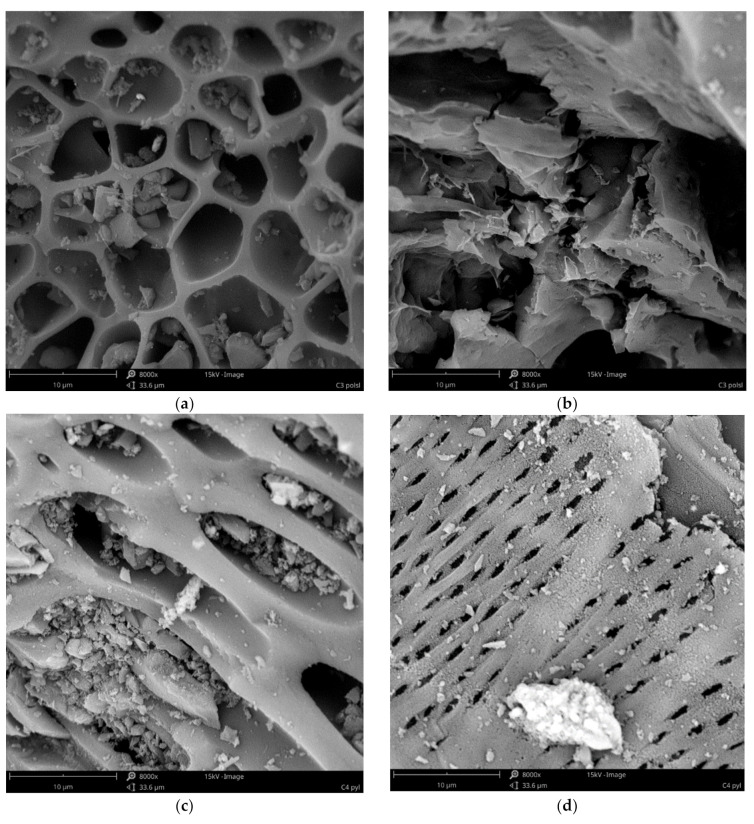

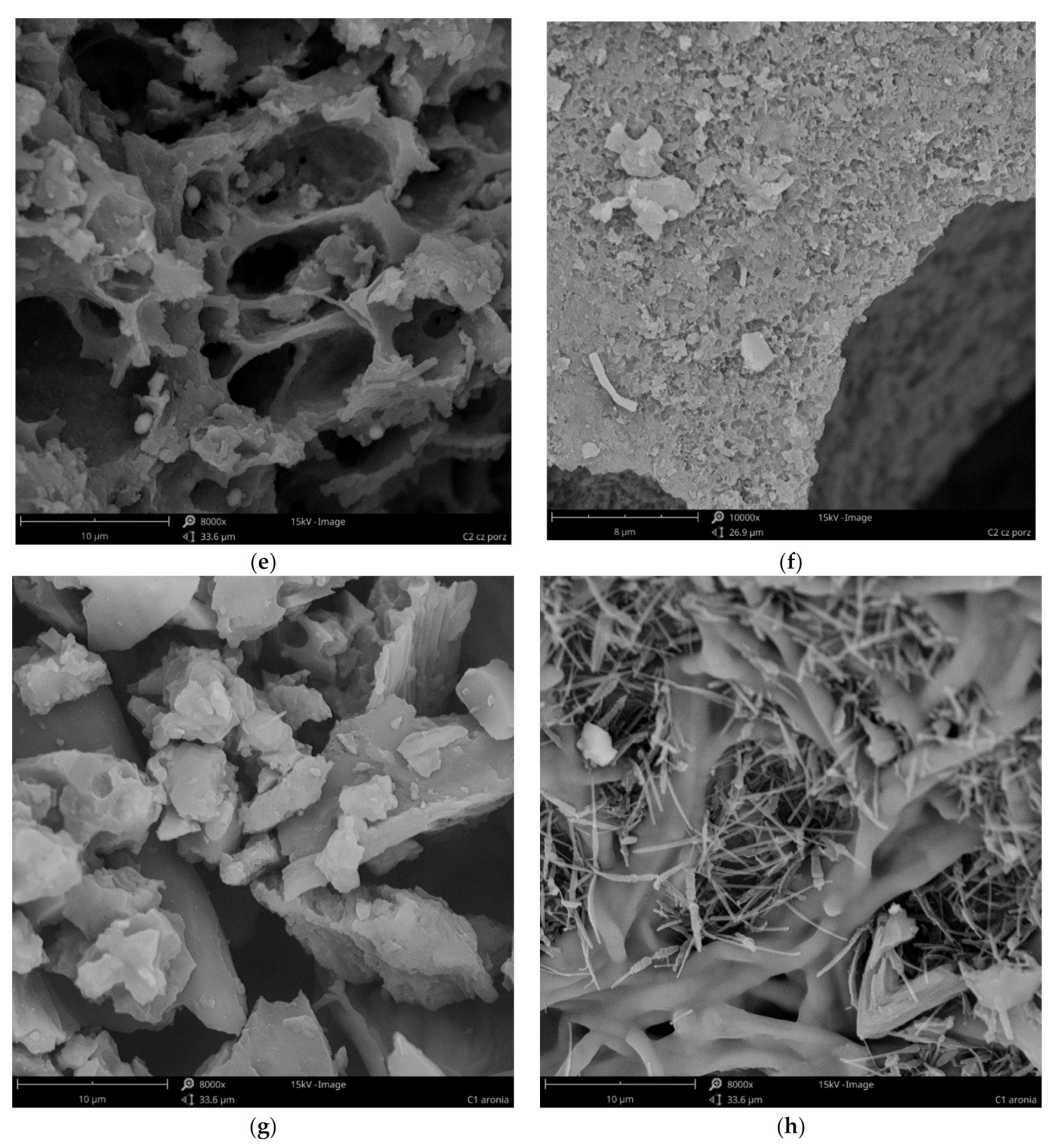
SEM micrographs of coals (magnification 8000× and 10000× (**f**)), (**a**,**b**)—C1 (active carbon of grain size 0.5–0.75 mm), (**c**,**d**)—C2 (pulverized activated carbon), (**e**,**f**)—C3 (carbonized blackcurrant pomace), (**g**,**h**)—C4 (carbonized chokeberry pomace).

The carbons used for the preparation of carbon-based sorbents prior to solvent loading were characterized by using scanning electron microscopy as SEM micrographs (Figure 1). The analysis has shown that not only do the studied coals differ from each other, but also different structures can occur within one type of coal (Figure 1a vs. Figure 1b, Figure 1c vs. Figure 1d, Figure 1e vs. Figure 1f and Figure 1g vs. Figure 1h). All materials were not homogeneous, and in all coals, there particles could be found with different porosities, compacted clods, or even fibrous structures. The material C1 (active carbon of grain size 0.5 ÷ 0.75 mm) is highly porous with grains full of visible pores with a diameter of 5 ÷ 11 µm with smooth walls (Figure 1a) and with grains of a structure of less dense walls, but also of a large surface area (Figure 1b). Pulverized activated carbon (C2) has smaller (Figure 1d) and larger (Figure 1c) pores with smooth (Figure 1c) and rougher (Figure 1d) walls and an elongated shape. Both materials seem to have well developed porosity and surface, so they should be very effective sorbent. Our results confirmed that both C1 and C2 were very effective in Ta removal, reaching an efficiency of approximately 97% removal. C3 coal (carbonized blackcurrant pomace) is much less porous. Although there were some particles with relatively high pores (Figure 1e), there was also a high content of more compact, rather non-porous clods (Figure 1f). The pore structure is slightly disturbed, presumably by crystals of inorganic compounds that may be present in incinerated plant material (Figure 1e). This structure suggests, then, a lower surface and sorption properties; however, the surface area of nonporous pieces seems to be well-developed (Figure 1f). This is rather illusory, because material C3 had the lowest efficiency in Ta removal (81%) of all carbons used. The least macroporous material appeared to be C4 carbon obtained from chokeberry pomace. It consists of lumps with a compact structure (Figure 1g) and a form similar to nanotubes or fibrous structures (Figure 1h). This material is also less porous than C1 and C2, but due to fibers, its surface should be more developed than C3, so it should have a better sorption efficiency from it. In fact, this material had a bit better efficiency in Ta removal than C3, but still lower than activated carbons C1 and C2. On the basis of these results, it can be concluded that C1 and C2 coals, due to their macroporous structure, are better candidates as carriers for organic solvent.

### 3.2. Preliminary Sorption Studies

The sorption properties of prepared solvent-impregnated coals (C1, C2, C3, C4) and some other MIBK-loaded sorption materials (S1, S2, S3, S4, S5) were preliminarily tested in batch studies under the same conditions (mass of sorbent: 3.5 g, volume of Ta and Nb solution (178 ppm of Ta per Nb: 50 mL, contact time: 24 h, and temperature: 22 ± 1 °C). The removal efficiency (E (%)) and separation factor (SF) for both elements studied were determined (Table 1) to indicate the best material for the column Ta from Nb separation.

The highest tantalum removal efficiency was determined using active coals-based materials (>97%). Very good Ta sorption was also observed on MIBK-modified strong base anion exchange resin as well as on MIBK-soaked PUR foam (96% and 95%, respectively). The element was hardly retained on solvent-impregnated strong acid cation exchange resin (only 3.4%).

Niobium, in contrast to tantalum, was not retained by any carbon-based materials (C1–C4), nor by MIBK-impregnated PUR (removal efficiency close to 0%). The highest niobium removal efficiency (74%) was determined using bentonite soaked with MIBK and moderate sorption was observed when using modified strong base anion exchange resin (41%).

The separation factor was then calculated to estimate the separation potential of the studied system towards Ta and Nb. For this purpose, when the results of the determination of niobium before and after contacting with MIBK-immersed material did not differ significantly (removal efficiency close to 0%), the value of maximal estimated niobium determination error (1.5%) as the niobium removal efficiency was used for the calculations. This enabled us to estimate the minimal possible separation factors for studied systems and their comparison. This standardization is essential; otherwise, in the case of materials that do not remove niobium (E_Nb_ endeavors to 0 thus SF endeavors to ∞), the comparison of their separation potential was impossible or could be seriously misleading if, based on calculations, the niobium removal efficiency of one material is 0.1% and for the second, 0.01%. In this case, and in practice, niobium is not extracted by both materials (E_Nb_ equals approx. 0%) and such a difference is the result of the unavoidable determination method error. However, the separation factor calculated for the second material will be almost 10 times higher than for the first material, which is misleading and incorrect.

Based on the calculations, the highest separation factor was determined for active coals-based materials (>2123). Thus, one of these materials was selected for column studies. The active carbon of greater grain size (C1) was selected as a material suitable for flow studies without risk of column clogging.

### 3.3. Separation of Tantalum from Niobium Using Fixed-Bed Column

The separation effectivity of tantalum from niobium fluoride complexes on MIBK-impregnated active carbon (C1) was determined in flow system studies. Solutions of various Nb:Ta weight ratios were pumped through fixed-bed columns with a constant flow rate of 0.2 mL/min. This flow rate was selected based on preliminary studies performed in the range of 0.2 ÷ 1 mL/min, which proved that this flow rate range is within the kinetic area and the flow rate of 0.2 mL/min is the best for Ta from Nb removal.

**Figure 2:**
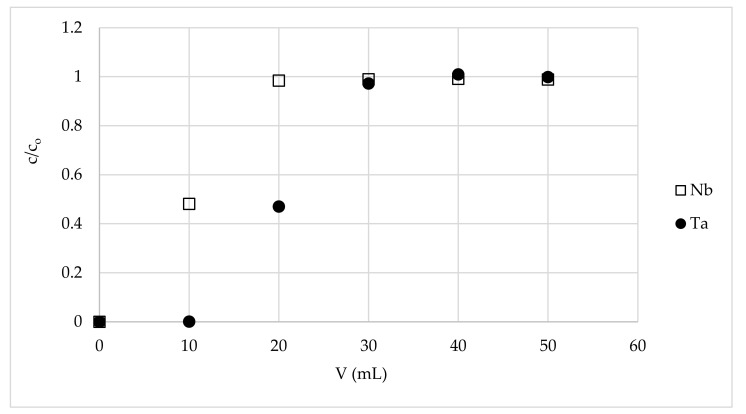
The breakthrough curves of Nb and Ta on a fixed-bed column with MIBK-impregnated active coal (m = 3.5 g, h = 18 cm, d = 0.8 cm, flow rate = 0.2 mL/min, feed solution Nb:Ta weight ratio = 1:1, temp. = 22 ± 1 °C).

**Figure 3:**
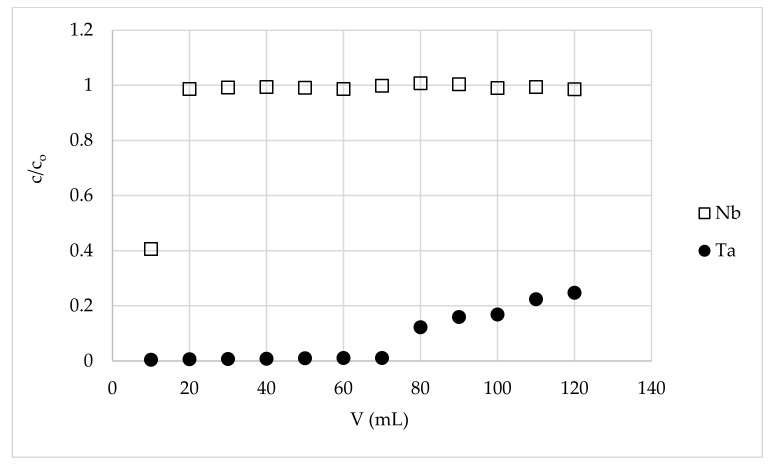
The breakthrough curves of Nb and Ta on a fixed-bed column with MIBK-impregnated active coal (m = 3.5 g, h = 18 cm, d = 0.8 cm, flow rate = 0.2 mL/min, feed solution Nb:Ta weight ratio = 100:1, temp. = 22 ± 1 °C).

**Figure 4:**
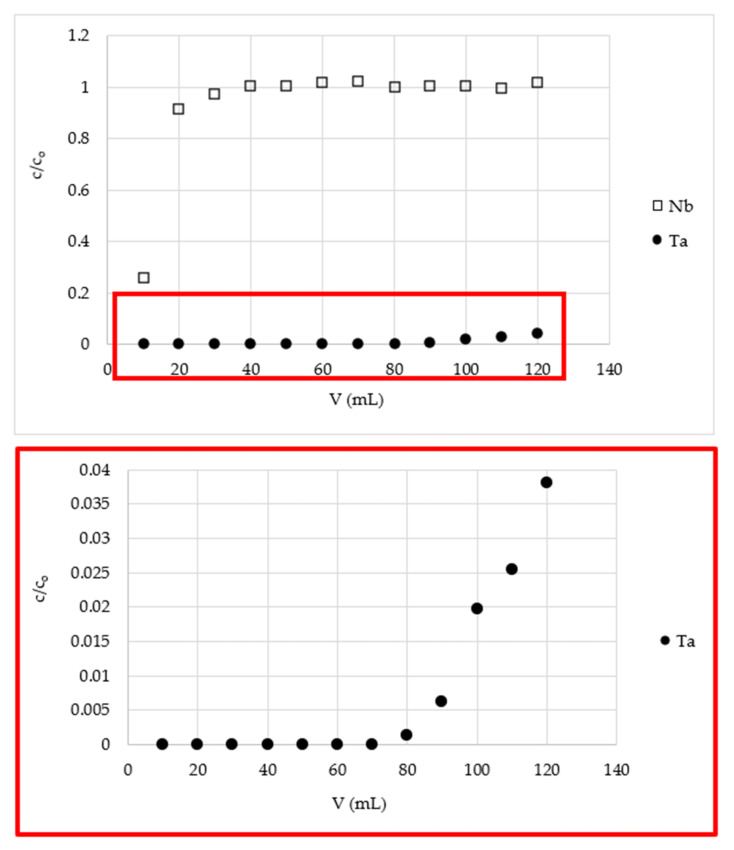
The breakthrough curves of Nb and Ta on a fixed-bed column with MIBK-impregnated active coal (m = 3.5 g, h = 18 cm, d = 0.8 cm, flow rate = 0.2 mL/min, feed solution Nb:Ta weight ratio = 1000:1, temp. = 22 ± 1 °C).

The results of tantalum separation from niobium are shown in the Figure 2, Figure 3 and Figure 4 and in Table 2. The breakthrough curves of niobium proved that this element was not retained on the tested material. The lower concentration of the metal ion in the first fraction is the result of the dilution of the feed solution with hydrofluoric acid solution (0.004%) that remained in the column among the bed grains after the pretreatment process. This decreases overall niobium yield in the process of its purification from tantalum, but it is inevitable and must be considered when the preparative system is calculated. However, in the proposed system, the niobium losses were not very large and were equal to 5÷10%, depending on the final product purity (Table 2). The breakthrough curves of tantalum proved to have a high affinity of tantalum ions for MIBK-impregnated active carbon, and thus high niobium from tantalum purification potential of the tested material. Tantalum operational capacity of the column used was different depending on the desired purity of niobium and the Nb:Ta ratio in the feed solution. It was equal to f.ex. 34, 1.7, and 0.16 mg/g for niobium purity and 320,000, 106, and 0.027 ppm Ta, obtained from a solution of Nb:Ta ratios 1:1, 100:1, and 1000:1, respectively. The most impressive result is obtaining 70 mL of a high purity niobium solution of tantalum content 0.027 ppm (in relation to Nb) with a yield of 88.4% niobium from a solution of 1000 ppm of tantalum in relation to the niobium content. This means that the purge factor was 35,000, which is a great achievement. As mentioned in the introduction, recently published papers mainly concern the separation of the elements from their ores and their subsequent separation, and the level of purification is not very deep. The highest reported purity of niobium is about 99.5% [43], 99% [18], 98.88% [40], or 87% [39]. In our studies, such material is only a starting material for the obtaining ultrapure niobium, so it is difficult to compare the cited results with ours.

### 3.4. Stripping of Tantalum from Solvent-Impregnated Active Coal

**Figure 5:**
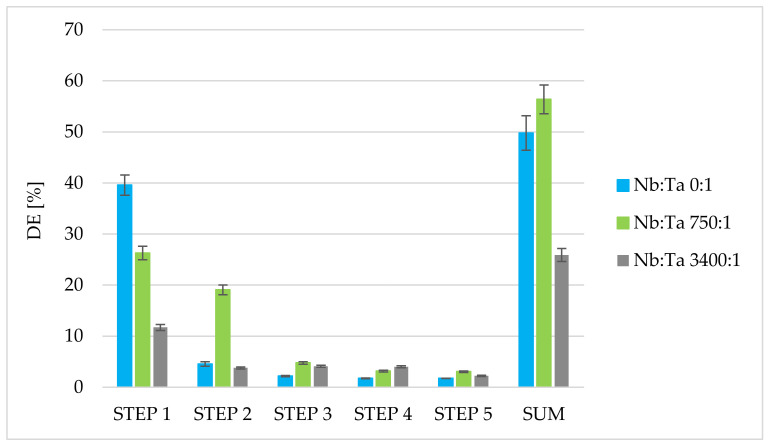
Desorption efficiency of various amounts of tantalum from MIBK-impregnated active coal using a 4% ammonium oxalate solution (m = 3 g, V = 40 mL, t = 15 min, temp. = 22 ± 1 °C, and number of steps: 5).

**Figure 6:**
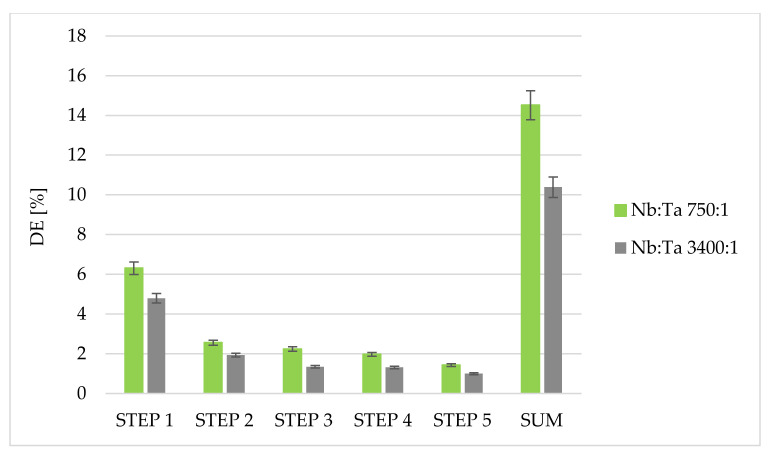
Desorption efficiency of various amounts of tantalum from MIBK-impregnated active coal using a 15% hydrogen peroxide solution (m = 3 g, V = 40 mL, t = 15 min, temp. = 22 ± 1 °C, number of steps: 5).

In a further study, it was examined whether it was possible to use the same portion of impregnated carbon multiple times to purify niobium from tantalum. For this purpose, desorption studies were performed in batch mode. The solutions of two various stripping agents, ammonium oxalate and hydrogen peroxide, which form stable complexes with tantalum ions, were utilized. By analogy with classical liquid-liquid extraction, it was assumed that tantalum is retained on the column in the immobilized MIBK as the (TaF_7_)^2−^ ion ((NbOF_5_)^2−^ ions coexisted with (TaF_7_)^2−^ ions in aqueous solution of low fluoride content and are not extracted into organic phase) [25]. Upon desorption (re-extraction) with ammonium oxalate solution, the (TaO(C_2_O_4_)_2_)^−^ ion is the most probable form [22,25], and upon desorption with hydrogen peroxide solution, the (Ta(O_2_)F_5_)^2−^ ion is the most probable form [44]. The desorption process was carried out in five steps, and the results are depicted in Figure 5 and Figure 6. Definitively better desorption efficiency was achieved in the case of using a 4% ammonium oxalate solution rather than a 15% hydrogen peroxide solution. Using the first stripping agent, about 50% of tantalum retained on MIBK-impregnated active coal may be recovered. Most of the tantalum is recovered in the first and second steps, so desorption was stopped at the fifth step, recognizing that continuing the process would not significantly improve the desorption percentage obtained. The desorption efficiency was also affected by the amount of tantalum retained on the active coal in the sorption process. Differences in the amount of tantalum retained on the resin in the batch sorption process resulted from the use of solutions with different ratios of niobium to tantalum while maintaining a comparable total concentration of Nb and Ta ions in solution. Lowering the amount of tantalum on MIBK-impregnated active coal (Nb:Ta 3400:1) resulted in a significant decrease in desorption efficiency. This may be due to the binding of some of the tantalum ions by the activated carbon (some sorption capacity of untreated activated carbon towards tantalum was confirmed in additional studies) and this effect (as constant for given carbon amount) can be more significant for desorption when the total amount of tantalum retained on the impregnated carbon is small. Additionally, the relative percentage of desorption for each step (related to the sum of desorption from all steps counted as 100% for the given Nb:Ta ratio) was calculated to be 79.5%, 9.1%, 4.4%, 3.5%, 3.5%; or 46.6%, 33.9%, 8.5%, 5.6%, 5.4%; or 45.3%, 14.6%, 15.9%, 15.5%, 8.7% for steps 1, 2, 3, 4, and 5; and the Nb:Ta ratios 0:1 or 750:1 or 3400:1, respectively. The percentage of tantalum removed in the first desorption step relative to the total tantalum desorbed in a given experiment (steps 1–5, i.e., 100%) is similar for smaller amounts of tantalum retained on MIBK-impregnated carbon (Nb:Ta 750:1 and 3500:1), and subsequent steps confirm that with decreasing amounts of tantalum on the sorbent, it is increasingly difficult to leach this element.

However, incomplete desorption is not a discriminating factor in the described method for purification of niobium from tantalum. It is a method that allows for the very deep purification of niobium (one step purge factor 35,000, niobium of tantalum content 0.027 ppm was obtained), which can lead to so-called fine chemicals that are obtained on a small scale. Therefore, the impregnated carbon used in the purification process can be disposed of by means of incineration with simultaneous recovery of tantalum, and it is an acceptable method. The dedication of our method to obtaining ultrapure reagents on a small scale also justifies the use of small amounts of HF, not recommended for safety reasons, whose presence provides such an excellent separation effect. It should be mentioned that in the recent literature, fluoride systems for the separation of Nb and Ta are still eagerly used [40,45], although gradually one can find attempts to eliminate the use of fluoride systems for the separation of Nb and Ta in oxalate [46,47] or phosphate [48] or electrochemical [49] systems, which is certainly a very good direction for large-scale industrial separation of these elements.

## 4. Conclusions

On the basis of results presented in this paper, we propose a new, efficient method for separating trace amounts of Ta(V) from Nb(V) using extraction chromatography with the use of active carbon impregnated with MIBK solvent. Our sorbent is inexpensive, easy to prepare, and may be applied in flow systems. It is selective for tantalum in the Nb–Ta fluoride complexes system and separation factor Ta/Nb in batch studies is greater than 2123. Dynamic studies using fixed-bed columns with MIBK-impregnated active carbon proved the possibility of obtaining a high purity niobium solution of tantalum content 0.027 ppm (in relation to Nb) with 88.4% yield of niobium from solution of Nb:Ta weight ratio 1000:1. This means that the purge factor was equal to 35,000 in one step, is a great achievement. Due to incomplete desorption, we propose impregnated carbon used in the purification process to be disposed by means of incineration with simultaneous recovery of tantalum as an acceptable method, considering the small scale of fine chemicals (very pure niobium compounds) obtained. In summation, the presented method is a promising proposition for a new separation system for the preparation of niobium compounds with a low content of tantalum. Such compounds are of great interest, especially for quality control in reactor-grade niobium steel production and for development of new separation methods, such as very pure niobium matrices, which are crucial for preparing matrix reference solutions, or CRM for analytical methods.

## Figures and Tables

**Table 1:** Removal efficiency and separation factor of tantalum and niobium on various MIBK-impregnated materials.

	Material Modified with MIBK	Removal Efficiency of Ta E_Ta_ (%)	Removal Efficiency of Nb E_Nb_ (%)	Separation Factor SF
C1	active carbon	>97	<1.5 *	>2123
C2	pulverized activated carbon	>97	<1.5 *	>2123
C3	carbonized blackcurrant pomace	81	<1.5 *	>280
C4	carbonized chokeberry pomace	85	<1.5 *	>372
S1	strong base anion exchange resin	96	41	35
S2	strong acid cation exchange resin	3.4	9.6	0.3
S3	bentonite	82	74	1.6
S4	cellulose ion exchanger	45	14	5.0
S5	polyurethane foam in lumps	95	<1.5 *	>1248

* The removal efficiency was close to 0%, but maximal estimated niobium determination error was taken into consideration.

**Table 2:** Tantalum from niobium separation effectivity on a fixed-bed column with MIBK-impregnated active coal.

Approx. Ta:Nb Ratio in Feed Solution (Accurate ppm of Ta in Relation to Nb)	Volume of Collected Effluent, mL	Nb Yield, %	The Content of Ta in Relation to Nb in Collected Effluent, ppm	Purge Factor
1:1 (994,490)	10	48.1	3156	315
	20	73.3	320,477	3.1
	30	81.9	585,130	1.7
1:100 (10,373)	70	90.9	106.0	97.9
	120	94.6	906.7	11.4
1:1000 (946)	70	88.4	**0.027**	**35,037**
	120	93.3	7.69	123

## Data Availability

All the data is available within the manuscript.
